# Clinical *Coxiella burnetii* infection in sable and roan antelope in South Africa

**DOI:** 10.4102/ojvr.v91i1.2151

**Published:** 2024-07-26

**Authors:** Wikus Wiedeman, Akorfa B. Glover, Johan Steyl, Jacques O’Dell, Henriette van Heerden

**Affiliations:** 1 Faculty of Veterinary Science, University of Pretoria, Onderstepoort, South Africa; 2 Department of Veterinary Tropical Diseases, Faculty of Veterinary Science, University of Pretoria, Onderstepoort, South Africa; 3 Department of Paraclinical Sciences, Faculty of Veterinary Science, University of Pretoria, Onderstepoort, South Africa; 4 Centre for Veterinary Wildlife Studies, Faculty of Veterinary Science, University of Pretoria, Onderstepoort, South Africa

**Keywords:** *Coxiella burnetii*, sable, roan, abortion, South Africa

## Abstract

Various zoonotic microorganisms cause reproductive problems such as abortions and stillbirths, leading to economic losses on farms, particularly within livestock. In South Africa, bovine brucellosis is endemic in cattle, and from 2013–2018, outbreaks of *Brucella melitensis* occurred in sable. *Coxiella burnetii*, the agent responsible for the zoonotic disease known as Q-fever and/or coxiellosis, also causes reproductive problems and infects multiple domestic animal species worldwide, including humans. However, little is known of this disease in wildlife. With the expansion of the wildlife industry in South Africa, diseases like brucellosis and coxiellosis can significantly impact herd breeding success because of challenges in identifying, managing and treating diseases in wildlife populations. This study investigated samples obtained from aborted sable and roan antelope, initially suspected to be brucellosis, from game farms in South Africa using serology tests and ruminant VetMAX™ polymerase chain reaction (PCR) abortion kit. The presence of *C. burnetii* was confirmed with PCR in a sable abortion case, while samples from both sable and roan were seropositive for *C. burnetii* indirect enzyme-linked immunosorbent assay (iELISA). This study represents the initial report of *C. burnetii* infection in sable and roan antelope in South Africa. Epidemiological investigations are crucial to assess the risk of *C. burnetii* in sable and roan populations, as well as wildlife and livestock in general, across South Africa. This is important in intensive farming practices, particularly as Q-fever, being a zoonotic disease, poses a particular threat to the health of veterinarians and farm workers as well as domestic animals.

**Contribution:**

A report of clinical *C. burnetii* infection in the wildlife industry contributes towards the limited knowledge of this zoonotic disease in South Africa.

## Introduction

*Coxiella burnetii* is an obligate intracellular Gram-negative coccobacillus bacterium with two morphological variants, a small cell variant (SCV) and a large cell variant (LCV) in infected host cells. The LCV replicates in host cells, while the SCV creates an off-host spore form that makes *C. burnetii* highly resistant to the environment (Arricau-Bouver & Rodolakis 2005; Maurin & Raoult 1999). Historically, *C. burnetii* was the only member of the genus *Coxiella* until the recent discovery of *Coxiella*-like endosymbionts (CLEs), which share 97% of their genome with *C. burnetii* (Noda, Munderloh & Kurtti 1997; Zhong 2012). *Coxiella*-like endosymbionts are classified into four clades namely A–D with *C. burnetii* belonging to clade A, which also includes CLEs associated with soft ticks (Duron et al. 2015). Initially reported in 1935 as ‘Query Fever’ (Q-fever) due to symptom overlap with other febrile illnesses (Cutler, Bouzid & Cutler 2007; Stoker & Marmion 1955), *Coxiella burnetii* is zoonotic and widely distributed, naturally infecting goats, sheep, cattle, parturient domestic dogs and cats (Brezina et al. 1973). More recently, wildlife and ticks have been reported as carriers of the bacterium (Szymanska-Czerwinska, Jodelko & Niemczuk 2019) with over 40 tick species identified as natural hosts for *C. burnetii* in the transmission of Q-fever (Knobel et al. 2013).

*Coxiella burnetii* infection typically occurs through the inhalation of aerosols, with infected animals often displaying minimal clinical signs, except for occurrences such as abortions at the end of the pregnancy, stillbirths, weak offspring, retained placenta, endometritis, infertility and reduced birth rates (Clemente et al. 2008). Once an animal is infected, the organism can localise in various tissues including the mammary glands, supramammary lymph nodes, placenta, amniotic fluid, urine, faeces and uterus. This facilitates its shedding in the environment during parturition as well as through milk and faeces, thereby enabling transmission to other animals and humans (García-Pérez et al. 2009).

Human clinical infection with *C. burnetii* is prevalent among individuals with specific occupations, including farmers, people who hunt and process kangaroos (unique to Australia), veterinary personnel, sheep shearers, laboratory or abattoir personnel, hide (tannery) workers, stockyard workers and animal transporters (Dupuis et al. 1987, Domingo et al. 1999). Humans become infected after inhaling aerosolised organisms or through mucous membranes coming into contact with infected droplets during parturition. A large portion of *C. burnetii* infections (up to 60%) are asymptomatic in humans (Frean & Bloomberg 2007), with acute presentations including fever, headache and atypical pneumonia with endocarditis as the major clinical presentation in chronic infection (Maurin & Raoult 1999). Alarmingly, the dispersal of organisms in dust particles stirred by the wind has been identified as an important epidemiological factor in spread (Clark & Magalhaes 2018). Evidence from livestock and humans suggests that aerosols are the primary mode of transmission among wild hosts (González-Barrio & Ruiz-Fons 2019).

The first documented case of Q-fever in humans in South Africa dates back to the 1950s, involving a 2-year-old European boy in the Transvaal province exhibiting symptoms such as high fever, coughing and vomiting (Gear et al. 1950). Approximately 20 years later, *C. burnetii* was isolated from placental tissues of sheep and cattle collected from various farms throughout South Africa in 1972 (Schutte et al. 1976). Seroprevalence studies in animals detected 7.8% (Gummow, Poerstamper & Herr 1987) and 1.9% (Matthewman et al. 1997) in cattle and cats, respectively, in South Africa. Additionally, 42.2% *C. burnetii* deoxyribonucleic acid (DNA) were detected with polymerase chain reaction (PCR) in ticks from dogs and cats collected from various provinces in South Africa. These ticks belonged to unknown species (52.0%), followed by 44.0% in *Rhipicephalus sanguineus* and lastly, 4.0% in *Haemaphysalis elliptica* (Mtshali et al. 2017). Communal farms adjacent to Kruger National Park in the wildlife-livestock interface have shown a prevalence of 38.0% seropositivity for *C. burnetii* in cattle (Adesiyun et al. 2020). Furthermore, *C. burnetii* seroprevalence rates of slaughtered cattle, sheep and pigs at Gauteng abattoirs were reported as 9.4%, 4.3% and 0.9% in cattle, sheep and pigs, respectively (Mangena et al. 2021), while serological and molecular *C. burnetii* prevalence in cattle on Limpopo province farms were 24.28% and 15.67%, respectively (Sadiki et al. 2023). Despite being reportable to the World Organisation for Animal Health (WOAH), *C. burnetii* is currently not classified as a notifiable disease in South Africa according to the *Animal Diseases Act (No 35 of 1984)*.

Given the growing wildlife industry in South Africa, the monitoring and conservation value of sable and roan antelope necessitates improved management and disease control to reduce losses. Roan antelopes (*Hippotragus equinus*, family Bovidae) have been reported to be non-clinically infected with a new *Babesia* species (Oosthuizen et al. 2009) and clinically infected with *Trypanosoma* (Konnai et al. 2008), theileriosis (Steyl et al. 2012) and anthrax (Clegg et al. 2007). The survival of roan, classified as a vulnerable species under the threatened species regulations of the *National Environmental Biodiversity Act 10 (2004)*, could be further threatened by an increasing number of abortions. Babesiosis caused by *Babesia* species (*B. irvinsmithi* and novel *Babesia* sp.) has been reported in sable antelope. Some cases presented benignly, while others caused widespread oedema and anaemia (McInnes et al. 1991, Oosthuizen et al. 2008). *Parelaphostrongylus tenuis* caused progressive hindlimb ataxia and weakness in captive sable (Nichols et al. 1986), while Peter et al. (1999) demonstrated that experimentally infected sable can be carriers of *Ehrlichia ruminantium* (heartwater) (Peter et al. 1999). Clinical disease of foot and mouth virus has been reported in sable, and infection persisted in experimentally infected sable and was transitory (Ferris et al. 1989). Detection of unknown *Anaplasma* sp. in sable has been reported. However, no clinical disease was observed (Kuttler 1984). Sable was reported to be brucellosis seropositive, with *B. melitensis* isolation from animals with abortion, acute hindquarter paresis, swollen hocks and swollen carpal joints (Godfroid et al. 2013; Glover et al. 2020). From 2013 to 2018 that includes this study, an increase in brucellosis outbreaks were reported in sable across South Africa (Glover et al. 2020). *Coxiella burnetii* infection was reported in a stillbirth of a captive-bred sable antelope in a zoo in Lisbon, Portugal (Clemente et al. 2008).

This study initially investigated the causal agent suspected to be brucellosis because of various brucellosis outbreaks in sable at the time of the investigation. The investigation used samples from aborted sable and roan (*Hippotragus* species) antelope from game farms in South Africa. The objectives included brucellosis serological testing of serum and milk samples using Rose Bengal and indirect ELISA (iELISA) tests. Subsequent to a necropsy examination of an aborted sable sample, the study incorporated the use of the ruminant VetMAX™ PCR abortion kit, which includes *C. burnetii* that was suspected. The study proceeded to test serum and milk samples submitted for brucellosis serological testing using *C. burnetii* iELISA test.

## Methodology

The samples and information date back to 2016–2018, with the report of abortions by various wildlife farmers. During which time increased brucellosis outbreaks were reported. Most cases were detected in the Limpopo province, where serum samples were sent for brucellosis testing (Glover et al. 2020). Samples were confidentially collected and submitted by multiple veterinarians. An abortion case from Farm 1 in the Western Cape province identified with PCR led to serological testing for *C. burnetii* antibodies in serum samples initially submitted for brucellosis testing, which included serum samples from case studies 2 to 4 farms in the Thabazimbi area, Limpopo province.

### Case studies

In *case study 1* on Farm 1, a farmer contacted his local veterinarian regarding abortions among four of his sables in the Western Cape province of South Africa in February 2017. The owner bought the sable from another sable breeder in the Limpopo province of South Africa. Other sable breeders that sourced sables from the latter also experienced abortions in their herds. Brucellosis was suspected as it has been reported in sable in South Africa from 2004 to 2018 with an increase in outbreaks occurring from 2013 to 2018 (Glover et al. 2020). Fresh foetal tissues were submitted to the Department of Paraclinical Sciences, Section of Pathology at the University of Pretoria for necropsy. Following histological examination and suspicion of intracellular bacterial infection, freshly collected specimens were submitted to Molecular Diagnostic Services Pty (ltd), South Africa for ruminant VetMAX™ PCR abortion panel targeting *Anaplasma phagocytophilum, Bovine Herpes Virus Type* 4, *Campylobacter fetus, Chlamydophila* spp., *Coxiella burnetii, Leptospira* spp., *Listeria monocytogenes* and *Salmonella* spp. Sera were submitted for brucellosis serology testing at the serology laboratory of the Department of Veterinary Tropical Diseases (DVTD) at the University of Pretoria (see serological tests below).

The cases from Farms 2 to 4 were in the Thabazimbi area, Limpopo province, South Africa. The second case study (*case study 2* from Farm 2) consisted of serum samples from four sables (three cows and one bull) part of a herd where some sable cows were aborted from Farm 2 in November 2017. On the same farm, serum samples were collected from roan with carpal swellings because of bursae/hygroma formation that has been described as typical for brucellosis infection in February 2018 (Godfroid et al. 2013). *Case study 3* on Farm 3 consisted of five serum samples collected from sables in January 2018, and *case study 4* from Farm 4 consisted of four serum samples collected from Farm 4 in June 2018. These samples were submitted for smooth *Brucella* and *Coxiella* sp. titre screening (see serological tests procedure below) at the serology laboratory of The Department of Veterinary Tropical Diseases at the University of Pretoria. None of the case study animals were vaccinated with brucellosis and/or coxiellosis vaccines. The serological samples of sable cases collected in Limpopo province were part of a research project with animal ethics approval from University of Pretoria and section 20 approval under the *Animal Disease Act 35* of 1984 from the Department of Agriculture, Land Reform and Rural Development, South Africa.

#### Serological tests

Blood samples were centrifuged for 10 min at 1000 revolutions per minute (rpm), and 2 mL aliquots were stored at −20 °C for serological analysis. To detect *C. burnetii* antibodies using a commercial iELISA, the CHEKIT Q-fever Antibody ELISA Test Kit (IDEXX Laboratories, United States) was used according to the manufacturer’s instructions, which detects the two antigenic forms, namely phase I and phase II antibodies to provide a cumulative serological outcome (Meadows et al. 2015). An index of the tested serum optical density to the difference between positive and negative controls was calculated (S/P%, where *s* = optical density of the sample – optical density of the negative control, and *p* = optical density of the positive control – optical density of the negative control). Serum samples with indices S/P% < 30% were considered negative, 30% < S/P% > 40% were considered suspect, whereas samples with indices S/P% > 40% were considered positive for C. burnetii antibodies. Samples were run in duplicates and averages of the S/P% results were taken.

*Rose Bengal test* (RBT) was done using 50 µL volume of commercial IDEXX *Brucella* antigen (Montpellier, France) stained with Rose Bengal and mixed with an equal volume of test serum and the mixture was agitated gently for 4 min at room temperature on a rocker. Agglutination was detected after 4 min, and any visible agglutination was regarded as positive for brucellosis as compared with the positive control (WOAH 2022). Indirect brucellosis iELISA by IDvet (IDVet Innovative Diagnostics, France) was used to test the serum according to the manufacturer’s instructions with a cut-off value of 80% to determine the antibody-positive status.

The commercial iELISA kits are multispecies and were tested for domestic livestock such as cattle, sheep, goats and pigs. The sensitivity and specificity are unknown and might not be adequate for wild ungulates such as sable and roan antelope. Brucellosis sable seropositive and seronegative samples were included with the commercial kit controls.

### Ethical considerations

Ethical clearance to conduct this study was obtained from the University of Pretoria Faculty of Veterinary Science Animal Ethics Committee (No. V042-16 and V042-16[A1]).

## Results

In case study 1, an intracellular bacterial infection such as *C. burnetii* was suspected as the cause of the abortion of sable in February 2017 based on observed lesions at necropsy that included histopathology. The ruminant VetMAX™ abortion panel PCR result confirmed *C. burnetii* as the causal agent from the aborted material from the sable antelopes and excluded *Anaplasma phagocytophilum, Bovine Herpes Virus Type* 4, *Campylobacter fetus, Chlamydophila* spp., *Leptospira* spp., *Listeria monocytogenes* and *Salmonella* spp.

In case study 2 from Farm 2 situated in Limpopo province, the serum samples of the sable and roan antelope were submitted for brucellosis testing in November 2017 and February 2018, respectively, and tested seronegative for brucellosis using RBT and iELISA. Based on the results from case study 1 testing positive for *C. burnetii,* the sable and roan serum samples from Farm 2 were tested for Q-fever using serology. The Q-fever iELISA detected *C. burnetii* antibodies in three of the four sable antelope on Farm 2 and two of the five roan antelope tested seropositive for Q-fever, and another was suspect using the iELISA from this farm (Table 1). The Q-fever iELISA detected *C. burnetii* antibodies in the positive roan samples (*n* = 17/30 and *n* = 17/20) with high S/P% of 335% and 61.4% as S/P% of > 40% is considered positive (Table 1).

**TABLE 1:** The IDEXX Q-fever indirect enzyme-linked immunosorbent assay results highlighted in red indicate the presence of *Coxiella burnetii* antibodies (S/P values) from roan (case study 2) and sable samples (case studies 2, 3 and 4) from three farms in Limpopo province that were all brucellosis seronegative.

Sable (S) and Roan (R) case numbers	Mean S/P value (%)	ELISA reaction
**Sable on Farm 2 case study 2**		
S17/02	−2.81	Negative
S17/127	108.37†	Positive†
S17/24	95.55†	Positive†
S17/01	61.72†	Positive†
**Roan on Farm 2 case study 2**		
R17/11	33.80‡	Suspect‡
R17/20	61.40†	Positive†
R17/30	335.30†	Positive†
R17/90	3.60	Negative
R17/60	9.40	Negative
**Sable on Farm 3 case study 3**		
S17/13	103.58†	Positive†
S17/31	167.09†	Positive†
S17/16	76.56†	Positive†
S17/15	46.36†	Positive†
S17/12	69.40†	Positive†
**Sable on Farm 4 case study 4**		
S17/05	138.00†	Positive†
S17/09	130.00†	Positive†
S17/21	133.00†	Positive†
S17/10	146.00†	Positive†
**Controls**		
*Brucella* negative sable	0.00	Negative
*Brucella* positive sable	12.00	Negative
Negative control	0.00	Negative
Positive control	100.00†	Positive†

ELISA, enzyme-linked immunosorbent assay; S/P, sample to positive.

† , S/P% > 40% was considered positive for *C. burnetii* antibodies;

‡ , S/P% > 30% and < 40% was considered suspect of *C. burnetii* antibodies.

In case study 3 from Farm 3 in Limpopo province, the ELISA detected *C. burnetii* antibodies in all five sable samples on Farm 3 in January 2018, and in case study 4 on Farm 4 in Limpopo province, four of the six sable samples from Farm 4 were detected in June 2018 (Table 1).

## Discussion

In this study, reproductive problems such as abortions were reported in sable and roan on wildlife farms in western and northern parts of South Africa. The study findings revealed the presence of *C. burnetii* infection in both sable and roan populations within the country. During necropsy examination, lesions suggestive of *C. burnetii* were observed and detected in aborted material from sable using ruminant VetMAX PCR while eliminating *Anaplasma phagocytophilum, Bovine Herpes Virus Type* 4, *Campylobacter fetus, Chlamydophila* spp., *Leptospira* spp., *Listeria monocytogenes* and *Salmonella* spp. but not *Brucella* sp. Antibodies against *C. burnetii* from sable and roan (*Hippotragus* species) antelope with abortion history were detected in serum samples that tested seronegative for brucellosis. Furthermore, seropositive cases for coxiellosis were identified in both sable and roan antelopes from the same farm (Farm 2) despite this farm testing negative for brucellosis. This finding suggests that the animals on Farm 2 had been exposed to the *C. burnetii* antigen, possibly spreading between the sable and roan herds within the farm.

There are limited reports of *C. burnetii* infection in different wildlife but currently includes reproductive failure in the waterbuck (*Kobus ellipsiprymnus*) and sable antelope (*Hippotragus niger*) (reported in a zoo in Portugal, Clemente et al. 2008), dama gazelle (*Nanger dama*) and water buffalo (*Bubalus bubalis*) and placentitis in the Pacific harbor seal (*Phoca vitulina richardsi*), Steller sea lion (*Eumetopias jubatus*) and red deer (*Cervus elaphus*) (González-Barrio & Ruiz-Fons 2019). Thus, there is evidence that the impact of this disease should not be underestimated, and the role wild hosts plays needs to be further investigated.

Q-fever or coxiellosis is a multi-host pathogen, which is mainly asymptomatic in animals (Angelakis & Raoult 2010, Maurin & Raoult 1999) but causes reproductive losses in livestock (Oporto et al. 2006; Van Asseldonk, Prins & Bergevoet 2015). As a zoonotic disease, it is important to identify the clinical signs associated with coxiellosis to limit unnecessary exposure to infective material. In pregnant animals, abortions and stillbirths are the main clinical signs, whereas in non-pregnant animals, the infection is virtually asymptomatic and thus not easily identified. Following abortion, shedding of *C. burnetii* occurs in vaginal mucus, faeces and milk, with shedding patterns varying among species (Plummer et al. 2018). In the case of infected *C. burnetii* sable on Farm 1, despite veterinarian advice, the antelopes remained on the farm in the western part of South Africa, resulting in abortion cases being reported during each breeding season. This case study and the others emphasise the organism’s role in causing abortions and reducing reproductive efficiency. On Farm 2, where coxiellosis seropositive sable and roan animals were reported, the serum from the roan cow had high antibody titres against *C. burnetii* compared to milk from the same roan cow that tested seronegative. A review of the literature by Gale et al. (2015) identified that risks from *C. burnetii* through milk consumption are lower compared to transmission via inhalation of aerosols from parturient products and livestock contact.

The presence of coxiellosis seropositive sable antelopes on different farms in the northern region of South Africa (Farms 2, 3 and 4) suggests that the entire herds on these farms may be infected. It is imperative to conduct testing and implement management strategies to prevent *C. burnetii* from spreading to other animals on the farms (wildlife and/or livestock), through airborne transmission and/or trade of breeding stock. Airborne transmission is a primary mode of new herd infection, as demonstrated by a spatial model presented by Pandit et al. (2016), which indicated that 92% of new infections were attributed to this route, with the remainder linked to trade in cattle herds. Given *C. burnetii’s* ability to aerosolise easily, a significant increase in the number of infected animals worldwide due to this disease is anticipated. South Africa, known for its diverse animal populations, often maintains domestic animals and wildlife in close proximity to one another. This proximity increases the risk of cross-infection between domestic and wild animals and *vice versa*, complicating disease control efforts, particularly concerning zoonotic diseases, that pose a threat to human health.

Asymptomatic carriers in both animals and humans account for the most infected individuals, complicating disease diagnosis and treatment (Maurin & Raoult 1999). Vaccinations have been developed for both animals and humans but are not commercially available. The vaccines protected calves, improved fertility and decreased shedding in previously infected animals (Roest et al. 2013). Furthermore, dairy goats vaccinated against *C. burnetii*, followed by a virulent challenge, were able to prevent abortions in the goats (Bontje et al. 2016). However, the vaccine manufacturing needs an appropriate BSL-3 facility and the diagnostic tests do not differentiate vaccinated animals from naturally infected animals (Roest et al. 2013). The role of wildlife as asymptomatic reservoirs for zoonotic diseases raises concerns as wildlife is normally not vaccinated. Infected animals are usually treated with antibiotics (especially tetracycline) (Arricau-Bouver & Rodolakis 2005; Frean & Blumberg 2007). However, there is little evidence of effective antibiotic response, rendering disease control within a herd challenging. Treating sables and other antelope is even more challenging and costly, as it necessitates chemical immobilisation for treatment, making it both expensive and impractical to administer frequently. Because of the zoonotic nature, uncertainty of antimicrobial effectiveness and threat of spreading to other animals, culling infected animals remains the most effective method of controlling the spread of the disease. However, the identification of true carriers of the disease remains challenging. This study and the limited literature on *C. burnetii* infection in wildlife highlight the need for research on the occurrence in livestock and wildlife in South Africa and the development of effective and practical disease control methods. Therefore, surveillance for this disease in livestock and wildlife should be prioritised to prevent it from becoming a serious problem in South Africa.

## Conclusion

This study provides the first documented evidence of *C. burnetii* infection in sable and roan antelope in South Africa, highlighting a significant zoonotic disease with implications for wildlife management and public health. The confirmation of *C. burnetii* in these antelope species highlight the need for epidemiological surveillance and targeted disease control measures within the expanding wildlife industry in South Africa. Given the zoonotic potential of *C. burnetii* and its ability to cause reproductive issues, the findings emphasise the importance of monitoring and managing this pathogen to safeguard animal health and minimise risks to especially veterinarians, farmers and farm workers. Future research should focus on comprehensive epidemiological assessments to determine the prevalence and impact of *C. burnetii* across diverse wildlife and livestock populations, informing strategies to mitigate the spread of this pathogen and protect both animal and human health.
